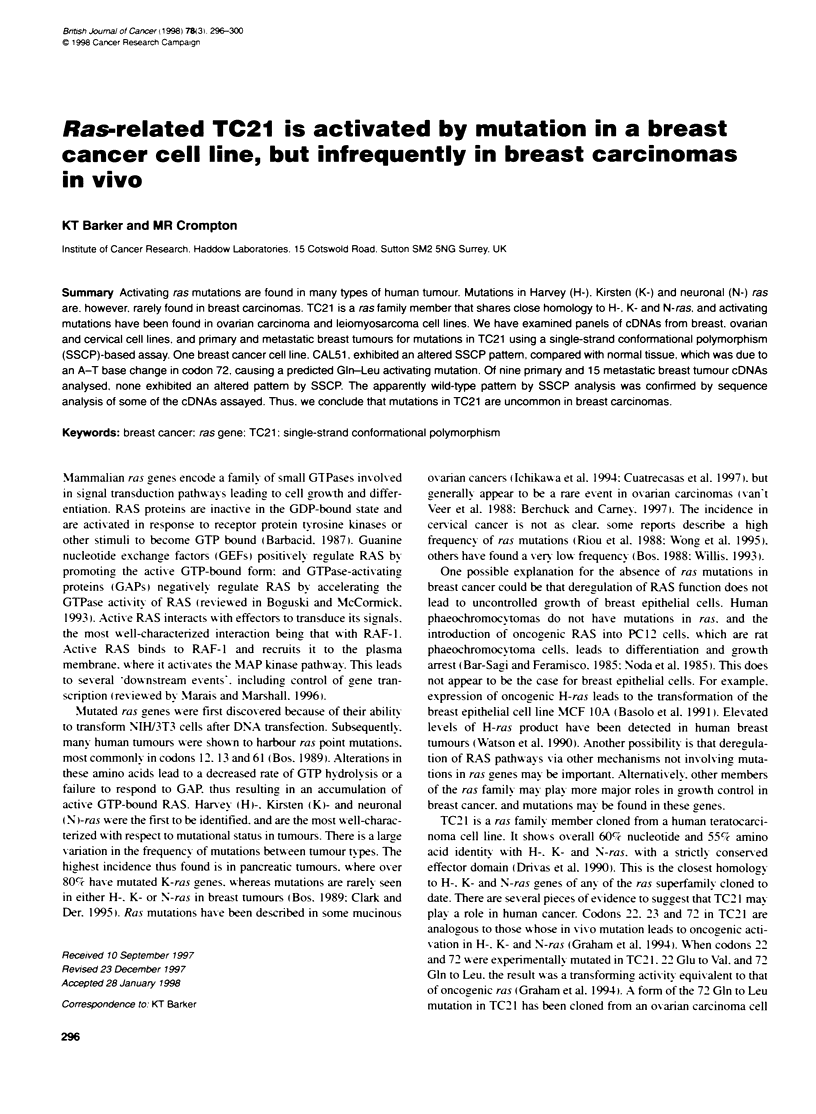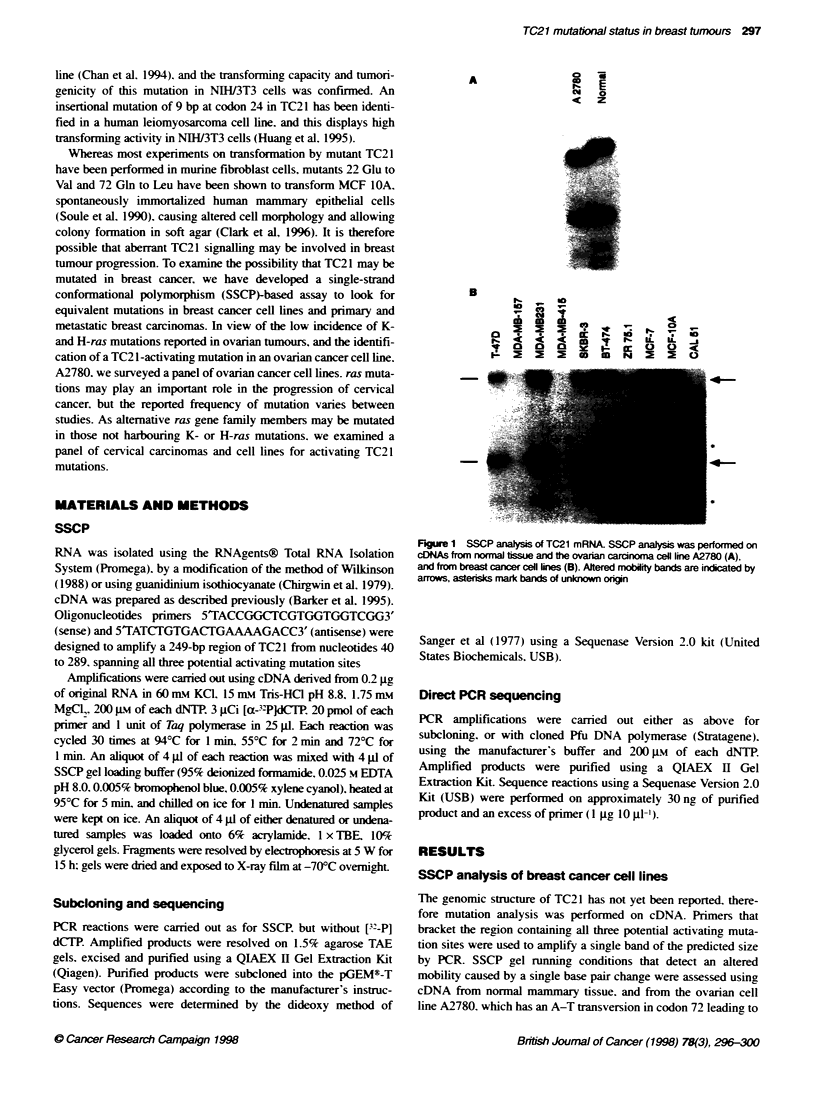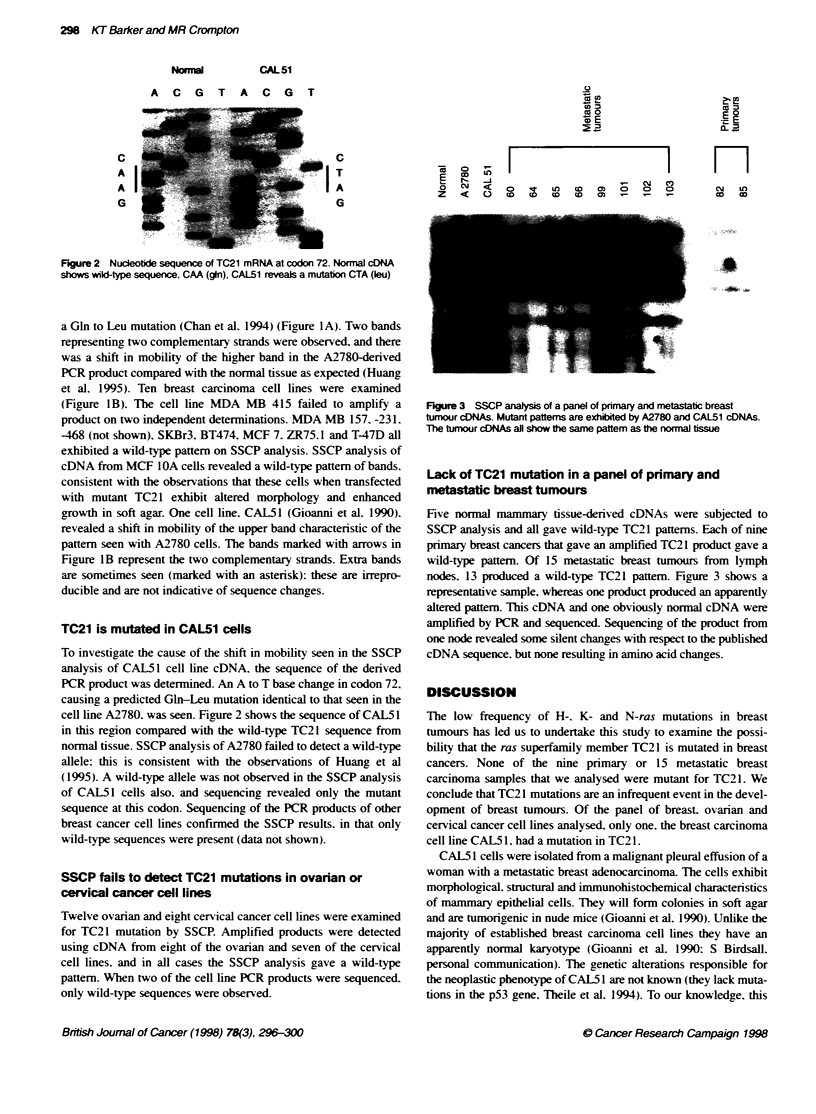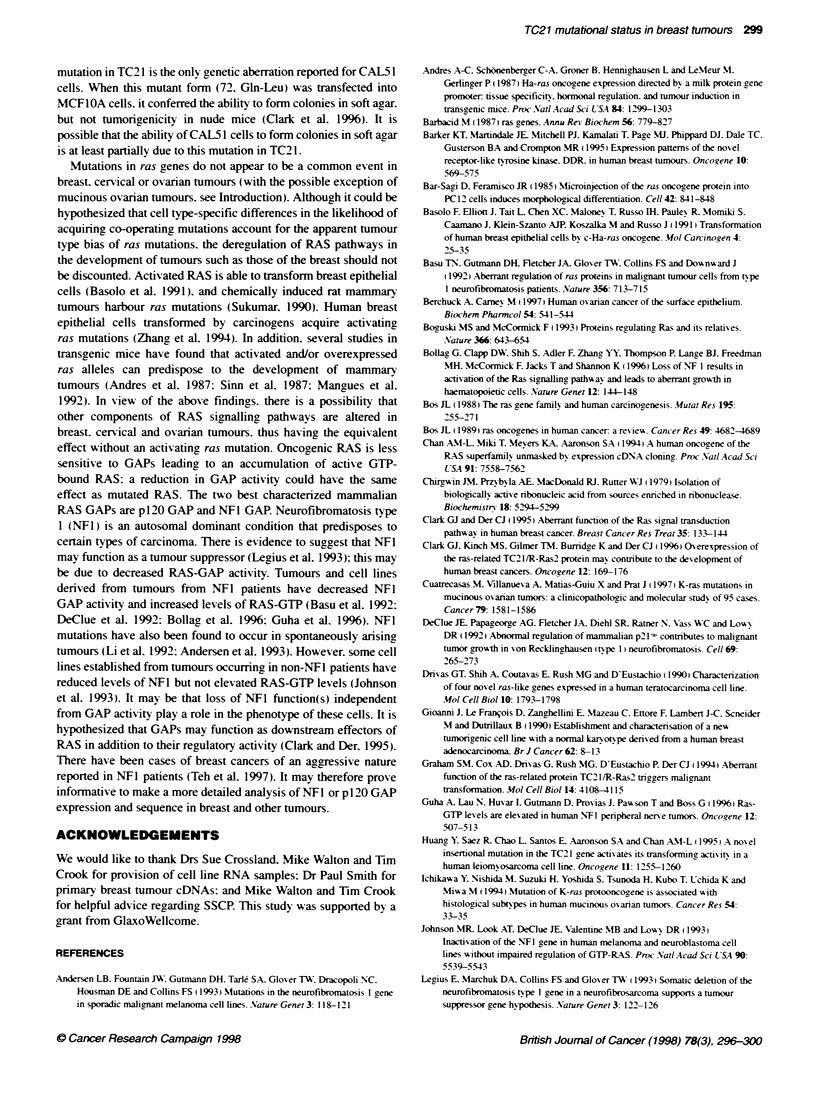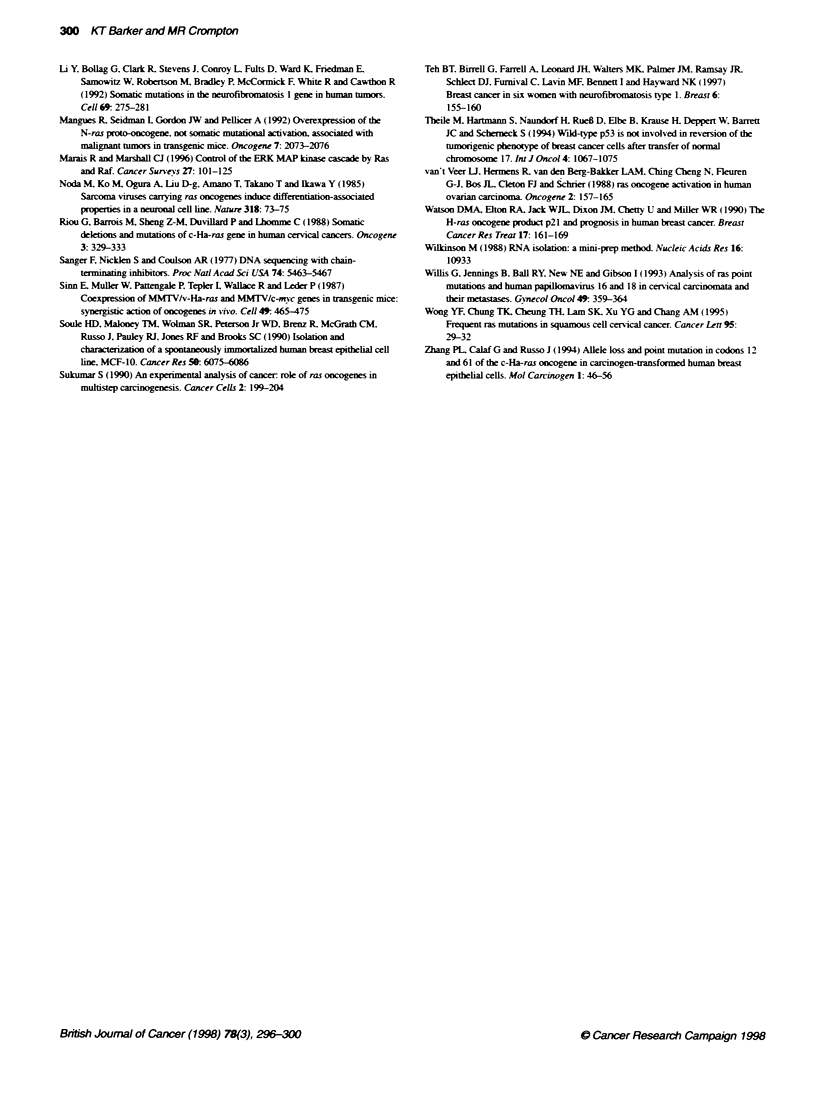# Ras-related TC21 is activated by mutation in a breast cancer cell line, but infrequently in breast carcinomas in vivo.

**DOI:** 10.1038/bjc.1998.490

**Published:** 1998-08

**Authors:** K. T. Barker, M. R. Crompton

**Affiliations:** Institute of Cancer Research, Haddow Laboratories, Sutton, Surrey, UK.

## Abstract

**Images:**


British Journal of Cancer (1998) 78(3). 296–300
© 1998 Cancer Research Campaign

### *Ras*-related TC21 is activated by mutation in a breast cancer cell line, but infrequently in breast carcinomas in vivo

**KT Barker and MR Crompton**

Institute of Cancer Research. Haddow Laboratories. 15 Cotswold Road. Sutton SM2 5NG Surrey. UK

**Summary** Activating *ras* mutations are found in many types of human tumour. Mutations in Harvey (H-). Kirsten (K-) and neuronal (N-) *ras* are. however. rarely found in breast carcinomas. TC21 is a *ras* family member that shares close homology to H-. K- and N-*ras*. and activating mutations have been found in ovarian carcinoma and leiomyosarcoma cell lines. We have examined panels of cDNAs from breast. ovarian and cervical cell lines. and primary and metastatic breast tumours for mutations in TC21 using a single-strand conformational polymorphism (SSCP)-based assay. One breast cancer cell line. CAL51. exhibited an altered SSCP pattern. compared with normal tissue. which was due to an A–T base change in codon 72. causing a predicted Gln–Leu activating mutation. Of nine primary and 15 metastatic breast tumour cDNAs analysed. none exhibited an altered pattern by SSCP. The apparently wild-type pattern by SSCP analysis was confirmed by sequence analysis of some of the cDNAs assayed. Thus. we conclude that mutations in TC21 are uncommon in breast carcinomas.

**Keywords:** breast cancer: *ras* gene: TC21: single-strand conformational polymorphism

Mammalian *ras* genes encode a family of small GTPases involved in signal transduction pathways leading to cell growth and differentiation. RAS proteins are inactive in the GDP-bound state and are activated in response to receptor protein tyrosine kinases or other stimuli to become GTP bound (Barbacid. 1987). Guanine nucleotide exchange factors (GEFs) positively regulate RAS by promoting the active GTP-bound form: and GTPase-activating proteins (GAPs) negatively regulate RAS by accelerating the GTPase activity of RAS (reviewed in Boguski and McCormick. 1993). Active RAS interacts with effectors to transduce its signals. the most well-characterized interaction being that with RAF-1. Active RAS binds to RAF-1 and recruits it to the plasma membrane. where it activates the MAP kinase pathway. This leads to several 'downstream events'. including control of gene transcription (reviewed by Marais and Marshall. 1996).

Mutated *ras* genes were first discovered because of their ability to transform NIH/3T3 cells after DNA transfection. Subsequently. many human tumours were shown to harbour *ras* point mutations. most commonly in codons 12. 13 and 61 (Bos. 1989). Alterations in these amino acids lead to a decreased rate of GTP hydrolysis or a failure to respond to GAP. thus resulting in an accumulation of active GTP-bound RAS. Harvey (H)-. Kirsten (K)- and neuronal (N)-*ras* were the first to be identified. and are the most well-characterized with respect to mutational status in tumours. There is a large variation in the frequency of mutations between tumour types. The highest incidence thus found is in pancreatic tumours. where over 80% have mutated K-*ras* genes. whereas mutations are rarely seen in either H-. K- or N-*ras* in breast tumours (Bos. 1989: Clark and Der. 1995). *Ras* mutations have been described in some mucinous ovarian cancers (Ichikawa et al. 1994: Cuatrecasas et al. 1997). but generally appear to be a rare event in ovarian carcinomas (van't Veer et al. 1988: Berchuck and Carney. 1997). The incidence in cervical cancer is not as clear. some reports describe a high frequency of *ras* mutations (Riou et al. 1988: Wong et al. 1995). others have found a very low frequency (Bos. 1988: Willis. 1993).

One possible explanation for the absence of *ras* mutations in breast cancer could be that deregulation of RAS function does not lead to uncontrolled growth of breast epithelial cells. Human phaeochromocytomas do not have mutations in *ras*. and the introduction of oncogenic RAS into PC12 cells. which are rat phaeochromocytoma cells. leads to differentiation and growth arrest (Bar-Sagi and Feramisco. 1985: Noda et al. 1985). This does not appear to be the case for breast epithelial cells. For example. expression of oncogenic H-*ras* leads to the transformation of the breast epithelial cell line MCF 10A (Basolo et al. 1991). Elevated levels of H-*ras* product have been detected in human breast tumours (Watson et al. 1990). Another possibility is that deregulation of RAS pathways via other mechanisms not involving mutations in *ras* genes may be important. Alternatively. other members of the *ras* family may play more major roles in growth control in breast cancer. and mutations may be found in these genes.

TC21 is a *ras* family member cloned from a human teratocarcinoma cell line. It shows overall 60% nucleotide and 55% amino acid identity with H-. K- and N-*ras*. with a strictly conserved effector domain (Drivas et al. 1990). This is the closest homology to H-. K- and N-*ras* genes of any of the *ras* superfamily cloned to date. There are several pieces of evidence to suggest that TC21 may play a role in human cancer. Codons 22. 23 and 72 in TC21 are analogous to those whose in vivo mutation leads to oncogenic activation in H-. K- and N-*ras* (Graham et al. 1994). When codons 22 and 72 were experimentally mutated in TC21. 22 Glu to Val. and 72 Gln to Leu. the result was a transforming activity equivalent to that of oncogenic *ras* (Graham et al. 1994). A form of the 72 Gln to Leu mutation in TC21 has been cloned from an ovarian carcinoma cell

*Received 10 September 1997*
*Revised 23 December 1997*
*Accepted 28 January 1998*

*Correspondence to:* KT Barker line (Chan et al, 1994), and the transforming capacity and tumorigenicity of this mutation in NIH/3T3 cells was confirmed. An insertional mutation of 9 bp at codon 24 in TC21 has been identified in a human leiomyosarcoma cell line, and this displays high transforming activity in NIH/3T3 cells (Huang et al, 1995).

Whereas most experiments on transformation by mutant TC21 have been performed in murine fibroblast cells, mutants 22 Glu to Val and 72 Gln to Leu have been shown to transform MCF 10A, spontaneously immortalized human mammary epithelial cells (Soule et al, 1990), causing altered cell morphology and allowing colony formation in soft agar (Clark et al, 1996). It is therefore possible that aberrant TC21 signalling may be involved in breast tumour progression. To examine the possibility that TC21 may be mutated in breast cancer, we have developed a single-strand conformational polymorphism (SSCP)-based assay to look for equivalent mutations in breast cancer cell lines and primary and metastatic breast carcinomas. In view of the low incidence of K- and H-*ras* mutations reported in ovarian tumours, and the identification of a TC21-activating mutation in an ovarian cancer cell line, A2780, we surveyed a panel of ovarian cancer cell lines. *ras* mutations may play an important role in the progression of cervical cancer, but the reported frequency of mutation varies between studies. As alternative *ras* gene family members may be mutated in those not harbouring K- or H-*ras* mutations, we examined a panel of cervical carcinomas and cell lines for activating TC21 mutations.

### MATERIALS AND METHODS

### SSCP

RNA was isolated using the RNAgents® Total RNA Isolation System (Promega), by a modification of the method of Wilkinson (1988) or using guanidinium isothiocyanate (Chirgwin et al, 1979). cDNA was prepared as described previously (Barker et al, 1995). Oligonucleotides primers 5′TACCGGCTCGTGGTGGTCGG3′ (sense) and 5′TATCTGTGACTGAAAAGACC3′ (antisense) were designed to amplify a 249-bp region of TC21 from nucleotides 40 to 289, spanning all three potential activating mutation sites

Amplifications were carried out using cDNA derived from 0.2 µg of original RNA in 60 mM KCl, 15 mM Tris-HCl pH 8.8, 1.75 mM $MgCl_2$, 200 µM of each dNTP, 3 µCi $[\alpha\text{-}^{32}P]$dCTP, 20 pmol of each primer and 1 unit of *Taq* polymerase in 25 µl. Each reaction was cycled 30 times at 94°C for 1 min, 55°C for 2 min and 72°C for 1 min. An aliquot of 4 µl of each reaction was mixed with 4 µl of SSCP gel loading buffer (95% deionized formamide, 0.025 M EDTA pH 8.0, 0.005% bromophenol blue, 0.005% xylene cyanol), heated at 95°C for 5 min, and chilled on ice for 1 min. Undenatured samples were kept on ice. An aliquot of 4 µl of either denatured or undenatured samples was loaded onto 6% acrylamide, 1 × TBE, 10% glycerol gels. Fragments were resolved by electrophoresis at 5 W for 15 h; gels were dried and exposed to X-ray film at –70°C overnight.

### Subcloning and sequencing

PCR reactions were carried out as for SSCP, but without $[^{32}\text{-}P]$ dCTP. Amplified products were resolved on 1.5% agarose TAE gels, excised and purified using a QIAEX II Gel Extraction Kit (Qiagen). Purified products were subcloned into the pGEM®-T Easy vector (Promega) according to the manufacturer's instructions. Sequences were determined by the dideoxy method of Sanger et al (1977) using a Sequenase Version 2.0 kit (United States Biochemicals, USB).

**Figure 1** SSCP analysis of TC21 mRNA. SSCP analysis was performed on cDNAs from normal tissue and the ovarian carcinoma cell line A2780 (**A**), and from breast cancer cell lines (**B**). Altered mobility bands are indicated by arrows, asterisks mark bands of unknown origin

### Direct PCR sequencing

PCR amplifications were carried out either as above for subcloning, or with cloned Pfu DNA polymerase (Stratagene), using the manufacturer's buffer and 200 µM of each dNTP. Amplified products were purified using a QIAEX II Gel Extraction Kit. Sequence reactions using a Sequenase Version 2.0 Kit (USB) were performed on approximately 30 ng of purified product and an excess of primer (1 µg 10 $\mu l^{-1}$).

### RESULTS

### SSCP analysis of breast cancer cell lines

The genomic structure of TC21 has not yet been reported, therefore mutation analysis was performed on cDNA. Primers that bracket the region containing all three potential activating mutation sites were used to amplify a single band of the predicted size by PCR. SSCP gel running conditions that detect an altered mobility caused by a single base pair change were assessed using cDNA from normal mammary tissue, and from the ovarian cell line A2780, which has an A–T transversion in codon 72 leading to

**Figure 2** Nucleotide sequence of TC21 mRNA at codon 72. Normal cDNA shows wild-type sequence, CAA (gln), CAL51 reveals a mutation CTA (leu)

a Gln to Leu mutation (Chan et al. 1994) (Figure 1A). Two bands representing two complementary strands were observed, and there was a shift in mobility of the higher band in the A2780-derived PCR product compared with the normal tissue as expected (Huang et al. 1995). Ten breast carcinoma cell lines were examined (Figure 1B). The cell line MDA MB 415 failed to amplify a product on two independent determinations. MDA MB 157, -231, -468 (not shown), SKBr3, BT474, MCF 7, ZR75.1 and T-47D all exhibited a wild-type pattern on SSCP analysis. SSCP analysis of cDNA from MCF 10A cells revealed a wild-type pattern of bands, consistent with the observations that these cells when transfected with mutant TC21 exhibit altered morphology and enhanced growth in soft agar. One cell line, CAL51 (Gioanni et al. 1990), revealed a shift in mobility of the upper band characteristic of the pattern seen with A2780 cells. The bands marked with arrows in Figure 1B represent the two complementary strands. Extra bands are sometimes seen (marked with an asterisk): these are irreproducible and are not indicative of sequence changes.

### TC21 is mutated in CAL51 cells

To investigate the cause of the shift in mobility seen in the SSCP analysis of CAL51 cell line cDNA, the sequence of the derived PCR product was determined. An A to T base change in codon 72, causing a predicted Gln–Leu mutation identical to that seen in the cell line A2780, was seen. Figure 2 shows the sequence of CAL51 in this region compared with the wild-type TC21 sequence from normal tissue. SSCP analysis of A2780 failed to detect a wild-type allele: this is consistent with the observations of Huang et al (1995). A wild-type allele was not observed in the SSCP analysis of CAL51 cells also, and sequencing revealed only the mutant sequence at this codon. Sequencing of the PCR products of other breast cancer cell lines confirmed the SSCP results, in that only wild-type sequences were present (data not shown).

### SSCP fails to detect TC21 mutations in ovarian or cervical cancer cell lines

Twelve ovarian and eight cervical cancer cell lines were examined for TC21 mutation by SSCP. Amplified products were detected using cDNA from eight of the ovarian and seven of the cervical cell lines, and in all cases the SSCP analysis gave a wild-type pattern. When two of the cell line PCR products were sequenced, only wild-type sequences were observed.

**Figure 3** SSCP analysis of a panel of primary and metastatic breast tumour cDNAs. Mutant patterns are exhibited by A2780 and CAL51 cDNAs. The tumour cDNAs all show the same pattern as the normal tissue

### Lack of TC21 mutation in a panel of primary and metastatic breast tumours

Five normal mammary tissue-derived cDNAs were subjected to SSCP analysis and all gave wild-type TC21 patterns. Each of nine primary breast cancers that gave an amplified TC21 product gave a wild-type pattern. Of 15 metastatic breast tumours from lymph nodes, 13 produced a wild-type TC21 pattern. Figure 3 shows a representative sample, whereas one product produced an apparently altered pattern. This cDNA and one obviously normal cDNA were amplified by PCR and sequenced. Sequencing of the product from one node revealed some silent changes with respect to the published cDNA sequence, but none resulting in amino acid changes.

### DISCUSSION

The low frequency of H-, K- and N-*ras* mutations in breast tumours has led us to undertake this study to examine the possibility that the *ras* superfamily member TC21 is mutated in breast cancers. None of the nine primary or 15 metastatic breast carcinoma samples that we analysed were mutant for TC21. We conclude that TC21 mutations are an infrequent event in the development of breast tumours. Of the panel of breast, ovarian and cervical cancer cell lines analysed, only one, the breast carcinoma cell line CAL51, had a mutation in TC21.

CAL51 cells were isolated from a malignant pleural effusion of a woman with a metastatic breast adenocarcinoma. The cells exhibit morphological, structural and immunohistochemical characteristics of mammary epithelial cells. They will form colonies in soft agar and are tumorigenic in nude mice (Gioanni et al. 1990). Unlike the majority of established breast carcinoma cell lines they have an apparently normal karyotype (Gioanni et al. 1990; S Birdsall, personal communication). The genetic alterations responsible for the neoplastic phenotype of CAL51 are not known (they lack mutations in the p53 gene, Theile et al. 1994). To our knowledge, this

© Cancer Research Campaign 1998

mutation in TC21 is the only genetic aberration reported for CAL51 cells. When this mutant form (72, Gln-Leu) was transfected into MCF10A cells, it conferred the ability to form colonies in soft agar, but not tumorigenicity in nude mice (Clark et al. 1996). It is possible that the ability of CAL51 cells to form colonies in soft agar is at least partially due to this mutation in TC21.

Mutations in *ras* genes do not appear to be a common event in breast, cervical or ovarian tumours (with the possible exception of mucinous ovarian tumours, see Introduction). Although it could be hypothesized that cell type-specific differences in the likelihood of acquiring co-operating mutations account for the apparent tumour type bias of *ras* mutations, the deregulation of RAS pathways in the development of tumours such as those of the breast should not be discounted. Activated RAS is able to transform breast epithelial cells (Basolo et al. 1991), and chemically induced rat mammary tumours harbour *ras* mutations (Sukumar, 1990). Human breast epithelial cells transformed by carcinogens acquire activating *ras* mutations (Zhang et al. 1994). In addition, several studies in transgenic mice have found that activated and/or overexpressed *ras* alleles can predispose to the development of mammary tumours (Andres et al. 1987; Sinn et al. 1987; Mangues et al. 1992). In view of the above findings, there is a possibility that other components of RAS signalling pathways are altered in breast, cervical and ovarian tumours, thus having the equivalent effect without an activating *ras* mutation. Oncogenic RAS is less sensitive to GAPs leading to an accumulation of active GTP-bound RAS; a reduction in GAP activity could have the same effect as mutated RAS. The two best characterized mammalian RAS GAPs are p120 GAP and NF1 GAP. Neurofibromatosis type 1 (NF1) is an autosomal dominant condition that predisposes to certain types of carcinoma. There is evidence to suggest that NF1 may function as a tumour suppressor (Legius et al. 1993); this may be due to decreased RAS-GAP activity. Tumours and cell lines derived from tumours from NF1 patients have decreased NF1 GAP activity and increased levels of RAS-GTP (Basu et al. 1992; DeClue et al. 1992; Bollag et al. 1996; Guha et al. 1996). NF1 mutations have also been found to occur in spontaneously arising tumours (Li et al. 1992; Andersen et al. 1993). However, some cell lines established from tumours occurring in non-NF1 patients have reduced levels of NF1 but not elevated RAS-GTP levels (Johnson et al. 1993). It may be that loss of NF1 function(s) independent from GAP activity play a role in the phenotype of these cells. It is hypothesized that GAPs may function as downstream effectors of RAS in addition to their regulatory activity (Clark and Der, 1995). There have been cases of breast cancers of an aggressive nature reported in NF1 patients (Teh et al. 1997). It may therefore prove informative to make a more detailed analysis of NF1 or p120 GAP expression and sequence in breast and other tumours.

### ACKNOWLEDGEMENTS

We would like to thank Drs Sue Crossland, Mike Walton and Tim Crook for provision of cell line RNA samples; Dr Paul Smith for primary breast tumour cDNAs; and Mike Walton and Tim Crook for helpful advice regarding SSCP. This study was supported by a grant from GlaxoWellcome.

### REFERENCES

Andersen LB, Fountain JW, Gutmann DH, Tarlé SA, Glover TW, Dracopoli NC, Housman DE and Collins FS (1993) Mutations in the neurofibromatosis 1 gene in sporadic malignant melanoma cell lines. *Nature Genet* **3**: 118–121

Andres A-C, Schönenberger C-A, Groner B, Hennighausen L and LeMeur M, Gerlinger P (1987) Ha-*ras* oncogene expression directed by a milk protein gene promoter: tissue specificity, hormonal regulation, and tumour induction in transgenic mice. *Proc Natl Acad Sci USA* **84**: 1299–1303

Barbacid M (1987) ras genes. *Annu Rev Biochem* **56**: 779–827

Barker KT, Martindale JE, Mitchell PJ, Kamalati T, Page MJ, Phippard DJ, Dale TC, Gusterson BA and Crompton MR (1995) Expression patterns of the novel receptor-like tyrosine kinase, DDR, in human breast tumours. *Oncogene* **10**: 569–575

Bar-Sagi D, Feramisco JR (1985) Microinjection of the *ras* oncogene protein into PC12 cells induces morphological differentiation. *Cell* **42**: 841–848

Basolo F, Elliott J, Tait L, Chen XC, Maloney T, Russo IH, Pauley R, Momiki S, Caamano J, Klein-Szanto AJP, Koszalka M and Russo J (1991) Transformation of human breast epithelial cells by c-Ha-*ras* oncogene. *Mol Carcinogen* **4**: 25–35

Basu TN, Gutmann DH, Fletcher JA, Glover TW, Collins FS and Downward J (1992) Aberrant regulation of *ras* proteins in malignant tumour cells from type 1 neurofibromatosis patients. *Nature* **356**: 713–715

Berchuck A, Carney M (1997) Human ovarian cancer of the surface epithelium. *Biochem Pharmcol* **54**: 541–544

Boguski MS and McCormick F (1993) Proteins regulating Ras and its relatives. *Nature* **366**: 643–654

Bollag G, Clapp DW, Shih S, Adler F, Zhang YY, Thompson P, Lange BJ, Freedman MH, McCormick F, Jacks T and Shannon K (1996) Loss of NF 1 results in activation of the Ras signalling pathway and leads to aberrant growth in haematopoietic cells. *Nature Genet* **12**: 144–148

Bos JL (1988) The ras gene family and human carcinogenesis. *Mutat Res* **195**: 255–271

Bos JL (1989) ras oncogenes in human cancer: a review. *Cancer Res* **49**: 4682–4689

Chan AM-L, Miki T, Meyers KA, Aaronson SA (1994) A human oncogene of the RAS superfamily unmasked by expression cDNA cloning. *Proc Natl Acad Sci USA* **91**: 7558–7562

Chirgwin JM, Przybyla AE, MacDonald RJ, Rutter WJ (1979) Isolation of biologically active ribonucleic acid from sources enriched in ribonuclease. *Biochemistry* **18**: 5294–5299

Clark GJ and Der CJ (1995) Aberrant function of the Ras signal transduction pathway in human breast cancer. *Breast Cancer Res Treat* **35**: 133–144

Clark GJ, Kinch MS, Gilmer TM, Burridge K and Der CJ (1996) Overexpression of the ras-related TC21/R-Ras2 protein may contribute to the development of human breast cancers. *Oncogene* **12**: 169–176

Cuatrecasas M, Villanueva A, Matias-Guiu X and Prat J (1997) K-ras mutations in mucinous ovarian tumors: a clinicopathologic and molecular study of 95 cases. *Cancer* **79**: 1581–1586

DeClue JE, Papageorge AG, Fletcher JA, Diehl SR, Ratner N, Vass WC and Lowy DR (1992) Abnormal regulation of mammalian p21$^{ras}$ contributes to malignant tumor growth in von Recklinghausen (type 1) neurofibromatosis. *Cell* **69**: 265–273

Drivas GT, Shih A, Coutavas E, Rush MG and D'Eustachio (1990) Characterization of four novel *ras*-like genes expressed in a human teratocarcinoma cell line. *Mol Cell Biol* **10**: 1793–1798

Gioanni J, Le François D, Zanghellini E, Mazeau C, Ettore F, Lambert J-C, Scneider M and Dutrillaux B (1990) Establishment and characterisation of a new tumorigenic cell line with a normal karyotype derived from a human breast adenocarcinoma. *Br J Cancer* **62**: 8–13

Graham SM, Cox AD, Drivas G, Rush MG, D'Eustachio P, Der CJ (1994) Aberrant function of the ras-related protein TC21/R-Ras2 triggers malignant transformation. *Mol Cell Biol* **14**: 4108–4115

Guha A, Lau N, Huvar I, Gutmann D, Provias J, Pawson T and Boss G (1996) Ras-GTP levels are elevated in human NF1 peripheral nerve tumors. *Oncogene* **12**: 507–513

Huang Y, Saez R, Chao L, Santos E, Aaronson SA and Chan AM-L (1995) A novel insertional mutation in the TC21 gene activates its transforming activity in a human leiomyosarcoma cell line. *Oncogene* **11**: 1255–1260

Ichikawa Y, Nishida M, Suzuki H, Yoshida S, Tsunoda H, Kubo T, Uchida K and Miwa M (1994) Mutation of K-*ras* protooncogene is associated with histological subtypes in human mucinous ovarian tumors. *Cancer Res* **54**: 33–35

Johnson MR, Look AT, DeClue JE, Valentine MB and Lowy DR (1993) Inactivation of the NF1 gene in human melanoma and neuroblastoma cell lines without impaired regulation of GTP-RAS. *Proc Natl Acad Sci USA* **90**: 5539–5543

Legius E, Marchuk DA, Collins FS and Glover TW (1993) Somatic deletion of the neurofibromatosis type 1 gene in a neurofibrosarcoma supports a tumour suppressor gene hypothesis. *Nature Genet* **3**: 122–126

Li Y, Bollag G, Clark R, Stevens J, Conroy L, Fults D, Ward K, Friedman E, Samowitz W, Robertson M, Bradley P, McCormick F, White R and Cawthon R (1992) Somatic mutations in the neurofibromatosis 1 gene in human tumors. *Cell* **69**: 275–281

Mangues R, Seidman I, Gordon JW and Pellicer A (1992) Overexpression of the N-*ras* proto-oncogene, not somatic mutational activation, associated with malignant tumors in transgenic mice. *Oncogene* **7**: 2073–2076

Marais R and Marshall CJ (1996) Control of the ERK MAP kinase cascade by Ras and Raf. *Cancer Surveys* **27**: 101–125

Noda M, Ko M, Ogura A, Liu D-g, Amano T, Takano T and Ikawa Y (1985) Sarcoma viruses carrying *ras* oncogenes induce differentiation-associated properties in a neuronal cell line. *Nature* **318**: 73–75

Riou G, Barrois M, Sheng Z-M, Duvillard P and Lhomme C (1988) Somatic deletions and mutations of c-Ha-*ras* gene in human cervical cancers. *Oncogene* **3**: 329–333

Sanger F, Nicklen S and Coulson AR (1977) DNA sequencing with chain-terminating inhibitors. *Proc Natl Acad Sci USA* **74**: 5463–5467

Sinn E, Muller W, Pattengale P, Tepler I, Wallace R and Leder P (1987) Coexpression of MMTV/v-Ha-*ras* and MMTV/c-*myc* genes in transgenic mice: synergistic action of oncogenes *in vivo*. *Cell* **49**: 465–475

Soule HD, Maloney TM, Wolman SR, Peterson Jr WD, Brenz R, McGrath CM, Russo J, Pauley RJ, Jones RF and Brooks SC (1990) Isolation and characterization of a spontaneously immortalized human breast epithelial cell line, MCF-10. *Cancer Res* **50**: 6075–6086

Sukumar S (1990) An experimental analysis of cancer: role of *ras* oncogenes in multistep carcinogenesis. *Cancer Cells* **2**: 199–204

Teh BT, Birrell G, Farrell A, Leonard JH, Walters MK, Palmer JM, Ramsay JR, Schlect DJ, Furnival C, Lavin MF, Bennett I and Hayward NK (1997) Breast cancer in six women with neurofibromatosis type 1. *Breast* **6**: 155–160

Theile M, Hartmann S, Naundorf H, Rueß D, Elbe B, Krause H, Deppert W, Barrett JC and Scherneck S (1994) Wild-type p53 is not involved in reversion of the tumorigenic phenotype of breast cancer cells after transfer of normal chromosome 17. *Int J Oncol* **4**: 1067–1075

van't Veer LJ, Hermens R, van den Berg-Bakker LAM, Ching Cheng N, Fleuren G-J, Bos JL, Cleton FJ and Schrier (1988) ras oncogene activation in human ovarian carcinoma. *Oncogene* **2**: 157–165

Watson DMA, Elton RA, Jack WJL, Dixon JM, Chetty U and Miller WR (1990) The H-*ras* oncogene product p21 and prognosis in human breast cancer. *Breast Cancer Res Treat* **17**: 161–169

Wilkinson M (1988) RNA isolation: a mini-prep method. *Nucleic Acids Res* **16**: 10933

Willis G, Jennings B, Ball RY, New NE and Gibson I (1993) Analysis of ras point mutations and human papillomavirus 16 and 18 in cervical carcinomata and their metastases. *Gynecol Oncol* **49**: 359–364

Wong YF, Chung TK, Cheung TH, Lam SK, Xu YG and Chang AM (1995) Frequent ras mutations in squamous cell cervical cancer. *Cancer Lett* **95**: 29–32

Zhang PL, Calaf G and Russo J (1994) Allele loss and point mutation in codons 12 and 61 of the c-Ha-*ras* oncogene in carcinogen-transformed human breast epithelial cells. *Mol Carcinogen* **1**: 46–56

© Cancer Research Campaign 1998